# Chromatin Accessibility Profiling of Keratinocytes from Clinically Healed Psoriatic Skin Reveals Epigenetic Alterations

**DOI:** 10.1016/j.xjidi.2025.100430

**Published:** 2025-10-28

**Authors:** Sayaka Shibata, Kentaro Awaji, Asumi Koyama, Haruka Taira, Toyoki Yamamoto, Lixin Li, Yukiko Ito, Shunsuke Miura, Takashi Yamashita, Takuya Miyagawa, Shinichi Sato

**Affiliations:** 1 Department of Dermatology, Graduate School of Medicine, The University of Tokyo, Tokyo, Japan

**Keywords:** ATAC-seq, Epigenetics, Keratinocytes, Psoriasis

## Abstract

Psoriasis is a chronic inflammatory skin disease with recurrence that often reappears at previously affected sites, suggesting an epigenetic imprint in healed skin. However, the chromatin landscape of clinically healed psoriatic keratinocytes remains uncharacterized. We performed Assay for Transposase-Accessible Chromatin using sequencing on epidermal keratinocytes isolated from psoriasis-affected, clinically healed, and control skin samples. Compared with those of healthy skin, psoriasis-affected keratinocytes exhibited widespread alterations in chromatin accessibility. Although most of these disease-associated changes resolved after clinical remission, 152 peaks remained differentially accessible, particularly at NF-κB–associated immune-regulatory loci, including *S100A7*/*A8/A9* and *IL36G*. Despite this residual chromatin accessibility, immunohistochemistry revealed a lack of detectable protein expression in healed keratinocytes, reflecting a transcriptionally poised but translationally inactive state. Pathway analysis revealed 2 recovery patterns: low-recovery peaks enriched for inflammatory pathways and high-recovery peaks linked to cytoskeletal remodeling and metabolism. These findings demonstrate that postremission keratinocytes retain a chromatin signature distinct from both affected and healthy skin. A limitation of this study is the absence of never-involved skin in assessing whether the chromatin changes identified are specific to previously affected skin. Nevertheless, this study provides insight into the molecular basis of disease memory that may underlie the skin’s susceptibility to relapse in psoriasis.

## Introduction

Psoriasis is a chronic skin disease characterized by recurrent inflammation and remission, profoundly impairing patients’ QOL (Armstrong and Read, 2020; Greb et al, 2016). Notably, psoriasis lesions often recur at previously affected sites (Gallais Sérézal et al, 2020, 2018), suggesting that postinflammatory skin retains a unique susceptibility to disease reactivation. Transcriptomic analyses such as RNA sequencing have been instrumental in elucidating gene expression and identifying critical pathways in diverse biological and pathological conditions. However, because they provide only a snapshot of transcriptional output, they offer limited insight into the upstream regulatory mechanisms that govern gene expression.

To address this limitation, the Assay for Transposase-Accessible Chromatin using sequencing (ATAC-seq) has emerged as a powerful method to profile open chromatin regions, offering insights into regulatory elements, including enhancers and promoters, which serve as a functional blueprint for transcription (Buenrostro et al, 2015; Corces et al, 2017; Grandi et al, 2022). In the context of psoriasis, ATAC-seq allows us to investigate chromatin accessibility dynamics during both active inflammation and clinical remission, offering a deeper understanding of postinflammatory regulatory changes. Although transcriptomic and chromatin accessibility analyses of nonlesional psoriatic whole skin have revealed altered gene expression and chromatin profiles compared with healthy skin (Gudjonsson et al, 2010; Keermann et al, 2015; Tang et al, 2021), it remains unclear how these changes manifest at the level of different cell types within the skin. Furthermore, whether similar alterations persist in clinically healed skin remains unresolved. Addressing these questions, our study isolates keratinocytes to dissect cell type–specific perspective on postremission epidermal regulation. Given their central role in psoriasis pathogenesis, understanding how keratinocyte chromatin landscapes are reshaped after clinical resolution will offer crucial insights into the postinflammatory state of the epidermis.

Inflammatory episodes can induce chromatin modifications that persist beyond the resolution of active disease. Such epigenetic reorganization may leave keratinocytes in a primed state, allowing them to respond more rapidly to future inflammatory stimuli (Netea et al, 2020; Tian and Lai, 2022). This phenomenon is reminiscent of trained immunity, originally described in innate immune cells, where prior inflammatory exposure enhances future responses through sustained chromatin accessibility changes (Bekkering et al, 2021; Mulder et al, 2019; Netea et al, 2020; Ochando et al, 2023). Although this phenomenon was initially thought to be restricted to immune cells, later studies revealed a similar regulatory process in epithelial cells, including keratinocytes (Bhargavi and Subbian, 2024; Cassone, 2018). A pioneering study demonstrated that inflammatory stimuli could induce persistent chromatin accessibility in mouse epidermal stem cells, even after the resolution of inflammation, thereby facilitating the rapid transcription of stress-response genes upon re-exposure (Larsen et al, 2021; Naik et al, 2017). Similarly, mechanical injury has been shown to induce altered chromatin landscape in mouse keratinocytes through chromatin remodeling, contributing to prolonged dermatitis (Shibata et al, 2020).

Building on these findings, we examine chromatin accessibility profiles of keratinocytes from psoriasis-affected, clinically healed, and control skin. We aim to characterize postremission chromatin landscapes in keratinocytes, providing a framework for understanding the preparatory epigenetic landscape that may shape future transcriptional responses in psoriasis.

## Results

### Key regulatory genes and pathways identified through differential chromatin accessibility in psoriasis-affected epidermal keratinocytes

**Figure 1:**
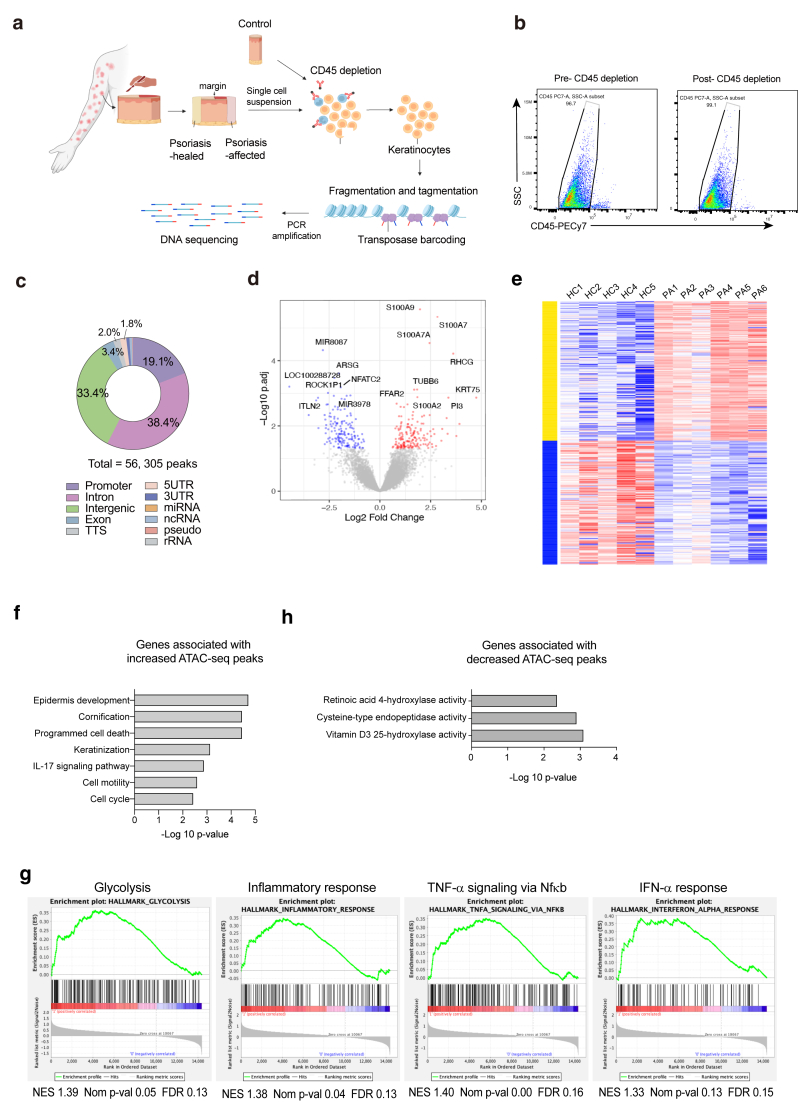
**Experimental workflow and key regulatory genes and pathways associated with chromatin accessibility dynamics in psoriasis-affected epidermal keratinocytes.(a)** Experimental workflow for this study. Psoriasis-affected and clinically healed skin samples were obtained from patients, and control skin was obtained from healthy individuals. CD45^+^ immune cells were depleted, followed by ATAC-seq and downstream analysis. **(b)** Flow cytometric analysis of CD45-negative cell proportion. CD45-negative cells in pre-CD45 depletion epidermal samples (left) and post-CD45 depletion epidermal samples (right) are shown. (**c)** Pie chart depicting genomic annotation of 56,305 chromatin accessible regions from 17 samples, categorized by genomic features. (**d)** Volcano plot showing the negative log10 of adjusted *P*-values (denoted as p.adj) against log2 fold changes of read counts (psoriasis-affected/control) for disease state differential ATAC-seq peaks within 3000 bp of the TSS. Red dots represent significantly increased peaks (adjusted *P* < .05) in psoriasis-affected samples, whereas blue dots represent significantly decreased peaks (adjusted *P* < .05). Key genes associated with differential ATAC-seq peaks are labeled. (**e)** Heatmap of K-means clustering of differential chromatin accessibility peaks located within 3000 bp from TSS in 6 psoriasis-affected (denoted as PA) and 5 HC epidermal keratinocytes. (**f)** Functional enrichment analysis of genes associated with increased disease-state ATAC-seq peaks in psoriasis-affected versus control keratinocytes. Y-axis: pathways; X-axis: −log10 *P*-value. (**g)** Functional enrichment analysis of genes associated with decreased disease-state ATAC-seq peaks in psoriasis-affected versus control keratinocytes. Y-axis: pathways; X-axis: −log10 *P*-value. (**h)** Gene set enrichment analysis comparing genes associated with increased disease-state ATAC-seq peaks with hallmark gene sets (H). The ranking metric scores (x-axis) are derived from log2 fold changes of read counts in psoriasis-affected compared with that in control keratinocytes. Enrichment profiles are presented, along with the NES, nominal *P*-value (denoted as Nom p-val), and FDR. ATAC-seq, Assay for Transposase-Accessible Chromatin using sequencing; FDR, false discovery rate; HC, healthy control; NES, normalized enrichment score; TSS, transcription start site.

This study investigated the chromatin landscape of epidermal keratinocytes in both psoriasis-affected skin, representing the disease state, and clinically healed skin, reflecting postremission status, in patients with recurrent skin symptoms (Figure 1a and b). The initial analysis revealed 111,166 chromatin peaks across all samples, of which 56,305 peaks had >10 counts after normalization. Most peaks were found in intergenic and intronic regions, with smaller fractions in promoter and exon regions (Figure 1c).

In the initial phase, we compared the chromatin landscape of epidermal keratinocytes from psoriasis-affected lesions with that from control skin, focusing on differentially accessible regions within 3000 bp from the transcription start site, where transcriptional regulatory elements are typically enriched (Boyle et al, 2008; Wang et al, 2012). Our analysis identified 127 regions with increased and 176 with decreased chromatin accessibility. The regulatory regions of key genes, including *S100A*s genes, keratin 75 gene *K75*, and *RHCG*, exhibited notable increases in chromatin accessibility, which may suggest a direct link to transcriptional modulation in psoriasis-affected keratinocytes. Conversely, decreased chromatin accessibility was noted in the regulatory regions of *NFATC2*, *ARSG*, and *MIR8087* (Figure 1d). K-means clustering identified genes linked to both increased (upper) and decreased (lower) altered chromatin accessibility, reflecting the intricate regulatory chromatin landscapes modulating psoriasis pathogenesis (Figure 1e). Enhanced chromatin accessibility regions were associated with keratinization, formation of the cornified envelope, IL-17 signaling pathway, cell cycle, epidermis development, positive regulation of NF-κB transcription factor activity, apoptotic signaling pathway, cell motility, and programmed cell death, indicating their pivotal roles in the pathological mechanisms of psoriasis at the chromatin level (Figure 1f). Gene set enrichment analysis (GSEA) further validated the induction of diverse functional pathways, including glycolysis, inflammatory response, and TNFα signaling through NF-κB as well as IFNα response (Figure 1g). Genes associated with decreased chromatin accessibility were enriched for pathways related to vitamin D3 and retinoic acid metabolism. Because both vitamin D analogs and systemic retinoids are clinically utilized for the treatment of psoriasis, these epigenetic changes may reflect disease-relevant therapeutic mechanisms (Figure 1h).

These identified pathways have already been reported and established through transcriptome analyses (Grine et al, 2015; Nestle et al, 2005). The findings from this study on chromatin accessibility extend these previous transcriptome-based insights, further clarifying the involvement of these genes and pathways at the chromatin level in the pathogenesis of psoriasis.

### Enhancer landscape alterations in psoriasis-affected epidermal keratinocytes

**Figure 2:**
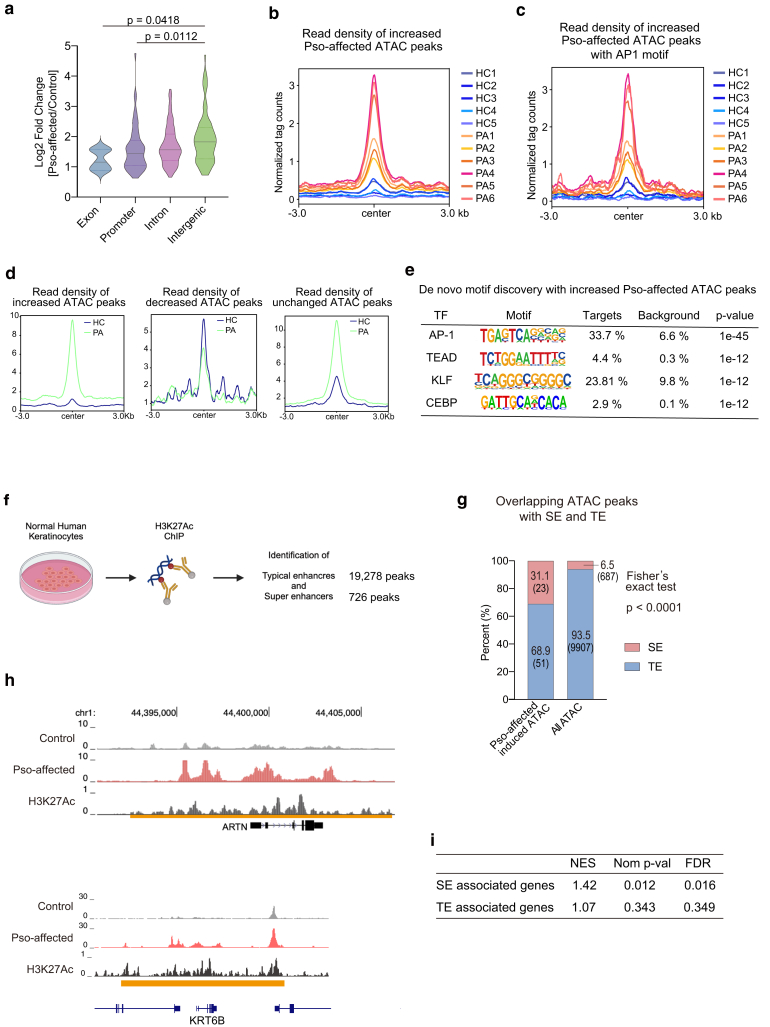
**Analysis of TF and enhancer dynamics in psoriasis-affected epidermal keratinocytes.(a)** Violin plots depicting the log2 fold changes of read counts (psoriasis affected/control) for differentially increased ATAC-seq peaks in psoriasis-affected versus control keratinocytes, categorized by genomic annotation (exon, promoter, intron, and intergenic). Statistical analysis was performed using 1-way ANOVA followed by Sidak's multiple comparisons test. (**b**, **c)** Histograms of normalized tag counts representing read density of individual samples centered on differentially increased ATAC-seq peaks in psoriasis-affected versus control keratinocytes. (**b)** All increased peaks. **(c)** Subset of increased peaks containing AP1 motifs. Each line represents an individual sample. HC denotes control, and PA denotes psoriasis affected. (**d)** Average tag density plots for significantly increased (left), significantly decreased (middle), and nonsignificantly unchanged (right) ATAC-seq peaks in psoriasis-affected versus control keratinocytes. (**e)** De novo motif (Homer) discovery analysis for differentially increased ATAC-seq peaks in psoriasis-affected versus control keratinocytes. The table lists the TF associated with identified motifs, the prevalence of these motifs in the target peaks and background, and the associated *P*-values. Pso-affected denotes psoriasis affected. (**f)** Experimental workflow for the identification of TEs and SEs in normal human keratinocytes using ChIP with H3K27Ac antibody. The analysis identified 19,278 TE and 726 SE peaks. (**g)** Bar chart depicting the proportion of enhancer-associated peaks in the SE and TE regions in the entire ATAC-seq peak dataset (right) and the subset of increased ATAC-seq peaks in psoriasis-affected versus control epidermal keratinocytes (left). Peaks located within 10,000 bp from TSS were analyzed. Fisher's exact test was used to compare the proportions, with a resulting *P* < .0001, indicating a significant difference. Pso-affected denotes psoriasis affected. (**h)** Genome Browser tracks on *ARTN* (upper) and *K6B* (lower) locus, depicting ATAC-seq peaks that are increased in psoriasis-affected keratinocytes (denoted as Pso-affected) versus control (control) derived from merged samples for each condition, along with ChIP sequencing for H3K27Ac in primary human keratinocytes. SE regions are marked with an orange line. (**i)** Results of gene set enrichment analysis comparing the genes associated with ATAC-seq peaks with the genes associated with SE and TE regions. Enrichment profiles are presented, along with the NES, nominal *P*-value (denoted as Nom p-val), and FDR. AP1, activator protein 1; ATAC-seq, Assay for Transposase-Accessible Chromatin using sequencing; ChIP, chromatin immunoprecipitation; FDR, false discovery rate; K6B, keratin 6B; NES, normalized enrichment score; SE, super-enhancer; TE, typical enhancer; TF, transcription factor; TSS, transcription start site.

Next, we expanded our focus to analyze regions with increased chromatin accessibility in psoriasis-affected keratinocytes, particularly within 10,000 bp of the transcription start site, distal regulatory elements that are critical for long-range transcriptional regulation (Boyle et al, 2008; Spitz and Furlong, 2012; Wang et al, 2012). Our analysis identified 264 with increased and 460 with decreased chromatin accessibility, as indicated by ATAC-seq peaks. Notably, alterations in chromatin accessibility, with statistically significant differences, were more pronounced in intergenic regions than in promoter and exon regions when comparing psoriasis-affected keratinocytes with controls (Figure 2a). These findings indicate a significant reprogramming of regulatory elements distal to promoters, suggesting the presence of functional transcriptional enhancers.

A consistent increase in read density compared with control keratinocytes was observed within the regions of increased chromatin accessibility across all psoriasis-affected keratinocytes (Figure 2b and c). Similar average plots for decreased and unchanged peaks confirmed overall signal consistency (Figure 2d). Regions with decreased accessibility showed lower signal intensity in psoriasis-affected keratinocytes, whereas unchanged peaks displayed only minor differences between groups, consistent with their lack of statistical significance. To identify transcription factors potentially involved in the increased chromatin accessibility in psoriasis-affected keratinocytes, we conducted de novo motif analysis on these regions. The analysis identified the activator protein 1 complex as the most significantly enriched motif with these regulatory sites, followed by significant enrichment of TEAD, KLF, and CEBP motifs (Figure 2c and e). The enrichment of activator protein 1 aligns with previous ATAC-seq studies exploring skin inflammatory or stress-responding conditions in mouse models and bulk human psoriatic skin (Naik et al, 2017; Shibata et al, 2020; Tang et al, 2021). The enrichment of TEAD, KLF, and CEBP motifs in this study suggests their potential involvement in keratinocyte-specific and chronic disease–associated regulatory programs.

We then focused on different types of enhancer regions, conventional typical enhancers and super-enhancers (SEs). SEs are distinguished by their high transcriptional activity and dense concentration of transcriptional machinery, constituting cell type–specific clusters critical for cellular identity and pathology (Pott and Lieb, 2015; Witte et al, 2015). We initially identified typical enhancers and SEs from H3K27Ac accumulation in normal human keratinocytes, where 726 and 19,278 peaks were detected, respectively (Figure 2f). Integrating these enhancer regions with ATAC-seq peaks that were significantly induced in psoriasis-affected keratinocytes revealed a remarkable distinction. Among all ATAC-seq peaks, the majority (93.5 %) were located in typical enhancers, and 6.5% were located at SEs. However, when focusing on ATAC-seq peaks significantly induced in psoriatic keratinocytes, >30% of peaks were located in SE domains (Figure 2g). Representative genes linked to SEs and induced ATAC-seq peaks included keratin K6B gene *K6B* and *ARTN*, which are known for their pivotal functions in keratinocytes (Edamitsu et al, 2019; Hidaka et al, 2017; Zhang et al, 2019) (Figure 2h). GSEA further provided the significance of these observations, showing that SE-associated genes were significantly enriched in the subset of genes with increased accessibility in psoriasis-affected keratinocytes (normalized enrichment score = 1.42, nominal *P* = .012, false discovery rate q-value = 0.016) compared with typical enhancer–associated genes (normalized enrichment score = 1.07, nominal *P* = .343, false discovery rate q-value = 0.349) (Figure 2i). This substantial enrichment of ATAC-seq peaks induced in psoriasis-affected keratinocytes may emphasize the potential of SE-mediated transcriptional reprogramming in the development of psoriasis.

### Selective persistence of chromatin accessibility and its regulatory signatures in clinically healed epidermal keratinocytes

**Figure 3:**
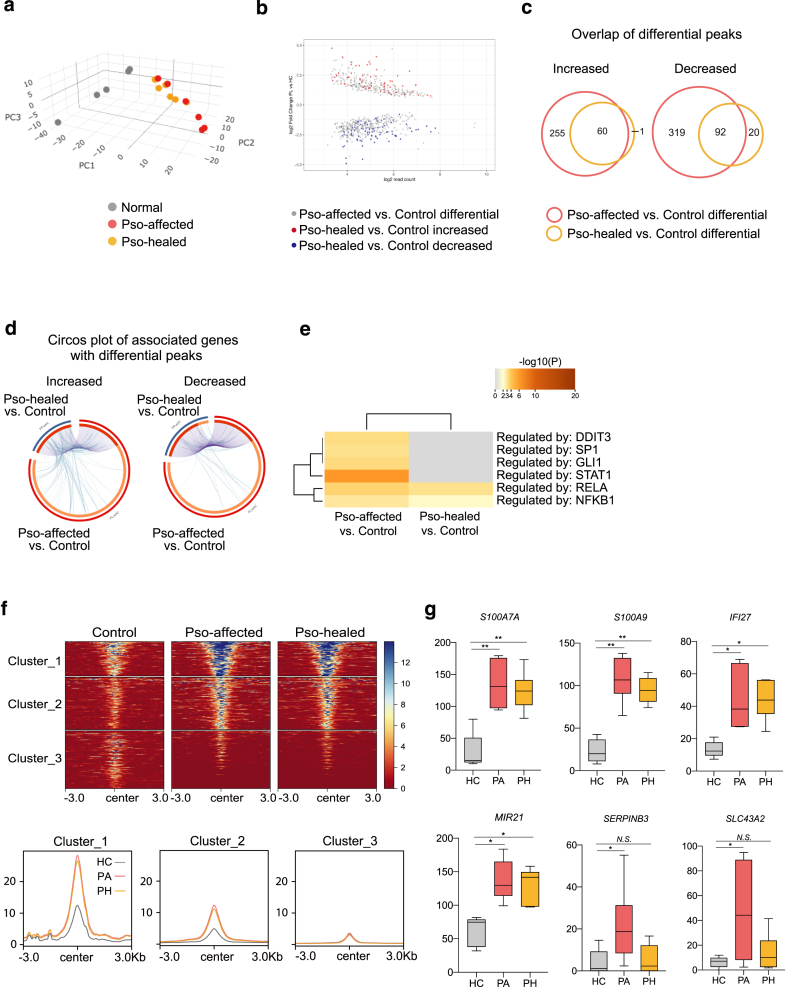
**Selective retention and regulatory characteristics of chromatin accessibility in clinically healed epidermal keratinocytes.(a)** PCA based on differentially accessible chromatin regions across all samples. Each point represents an individual sample: healthy control (gray), psoriasis affected (red), and psoriasis healed (orange). (**b)** MA plots displaying the log ratio versus the mean average of chromatin accessibility read counts in disease-state (denoted as Pso-affected) and clinically healed (denoted as Pso-healed) keratinocytes compared with that in control keratinocytes. Gray dots represent peaks that are significantly altered in disease-state (Pso-affected) keratinocytes. Red and blue dots indicate the subset of peaks with differential accessibility that remains significantly altered in clinically healed (Pso-healed) keratinocytes (red: increased; blue: decreased). **(c)** Venn diagram depicting the overlap of differentially accessible chromatin regions between disease-state (Pso-affected) and clinically healed (Pso-healed) keratinocytes compared with that in control keratinocytes. A total of 152 peaks (60 increased, 92 decreased) remained differentially accessible in healed keratinocytes, whereas 574 peaks (255 previously increased, 319 previously decreased) lost statistical significance after remission. (**d)** Circos plot depicting overlaps in genes associated with significantly (adjusted *P* < .05) increased (left) or decreased (right) ATAC-seq peaks. Comparisons include disease-state psoriasis-affected and clinically healed psoriasis-healed keratinocytes relative to control keratinocytes. Links represent shared genes between the 2 comparisons. (**e)** Enrichment analysis in TRRUST of genes associated with ATAC-seq peaks significantly (adjusted *P* < .05) increased in psoriasis-affected and psoriasis-healed versus in control keratinocytes. (**f)** Heatmap showing K-means clustering of ATAC-seq datasets from control, psoriasis-affected, and psoriasis-healed keratinocytes, centered on significantly (adjusted *P* < .05) increased ATAC-seq peaks containing RELA motifs identified in psoriasis-affected versus in control keratinocytes. Below, histograms display read densities within ±3.0 kb for the 3 clusters (cluster_1, cluster_2, and cluster_3), with control (HC), psoriasis-affected (denoted as PA), and psoriasis-healed (denoted as PH) samples shown separately. (**g)** Box plots showing normalized read counts in the gene loci of representative genes. Statistical significance is indicated as follows: ∗∗adjusted *P* < .05 and ∗adjusted *P* < .05. N.S. denotes not significant. Normalized read counts and adjusted *P*-values were calculated using DESeq2. ATAC-seq, Assay for Transposase-Accessible Chromatin using sequencing; HC, healthy control; PCA, principal component analysis.

The focus of our next investigation shifts to clinically healed skin lesions. Although the ideal control might be nonlesional skin from the same patients, such samples were not available in this study. Nonetheless, comparing chromatin accessibility in healed and affected keratinocytes with that in healthy skin keratinocytes remains valuable for assessing how disease state chromatin changes are maintained after clinical remission. To assess the global structure and variability within and between sample groups, we first performed principal component analysis on the basis of differentially accessible chromatin regions. This analysis showed that psoriasis-affected and psoriasis-healed keratinocytes clustered closely together, whereas healthy controls exhibited greater interindividual dispersion (Figure 3a). Next, to evaluate the retention or resolution of chromatin accessibility changes, we first visualized differential accessible peaks using MA plots. By overlaying differentially accessible peaks from psoriasis-affected versus control comparisons with those from healed versus control comparisons, we observed widespread chromatin accessibility alterations in disease-state keratinocytes (gray dots [Figure 3b]). Most of these changes lost statistical significance in clinically healed keratinocytes; a subset of regions retained altered accessibility (red and blue [Figure 3b]), indicative of selective maintenance of epigenetic modifications. A Venn diagram further illustrated these patterns, revealing that 152 peaks remained differentially accessible in healed keratinocytes (60 increased, 92 decreased), whereas 574 peaks lost statistical significance (255 previously increased, 319 previously decreased) (Figure 3c). The emergence of new peaks was minimal, with only 1 newly increased peak in healed keratinocytes, whereas decreased peaks showed greater dynamism, with 20 newly decreased peaks identified (Figure 3c).

To evaluate the potential impact of differences in these chromatin accessibility changes on gene expression, we examined the genes mapped to altered chromatin regions. We observed a significant overlap in genes associated with differential ATAC-seq peaks between disease-state psoriasis-affected and clinically healed keratinocytes relative to control keratinocytes, where both disease-state and healed keratinocytes shared a significant proportion of differentially accessible gene loci (Figure 3d). Subsequently, we performed an enrichment analysis using the TRRUST database within the Metascape framework to identify transcription factors regulating these genes. The analysis identified significant enrichment of genes regulated by transcription factors of NFKB1 and RELA in persisting differentially accessible regions (Figure 3e). We then extracted peaks containing RELA motifs that were significantly induced in psoriasis-affected keratinocytes. Overlaying these peaks with data from healed keratinocytes revealed that RELA-associated chromatin accessibility was largely maintained after clinical remission (Figure 3f). Representative examples of these signatures included genes critical to skin inflammation and immune responses, such as *S100A7, S100A8, S100A9*, and *IL36G*, which are well-known for their roles in the development and maintenance of psoriasis (Figure 3g).

Although our primary comparisons focused on psoriasis-affected and psoriasis-healed samples relative to healthy controls, we also directly compared chromatin accessibility between psoriasis-affected and psoriasis-healed keratinocytes. This pairwise comparison did not yield statistically significant differences in overall accessibility patterns (adjusted *P* > .05), likely reflecting the use of patient-matched samples. Nevertheless, substantial differences remained between both psoriasis-affected and psoriasis-healed keratinocytes relative to healthy controls, suggesting that clinical resolution does not fully normalize the chromatin landscape. Taken together, these findings suggest that selective retention at immune-regulatory loci may constitute an epigenetic basis for site-specific disease recurrence.

### Absence of protein expression despite sustained chromatin accessibility in clinically healed epidermis

**Figure 4:**
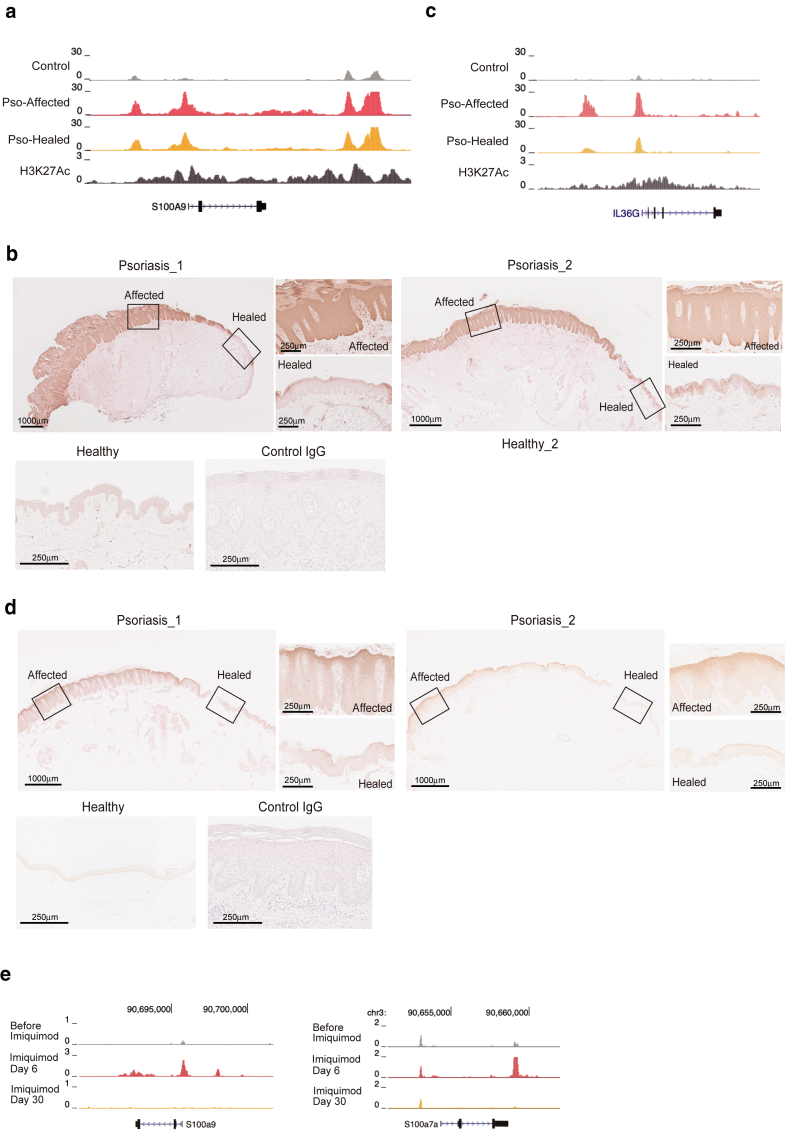
**Residual chromatin accessibility does not translate into protein expression in clinically healed epidermis.(a)** Genome Browser tracks on human *S100A9* loci, depicting chromatin accessibility peaks in disease-state (denoted as Pso-affected), clinically healed (denoted as Pso-healed), and control (denoted as control) keratinocytes, derived from merged samples for each condition. Increased chromatin accessibility is observed in disease-state keratinocytes and remains detectable in clinically healed keratinocytes. ChIP sequencing for H3K27Ac in primary human keratinocytes is also shown, indicating active regulatory regions. (**b)** Immunohistochemistry for S100A9 and isotype control in psoriasis-affected and psoriasis-healed lesions (upper panels: Psoriasis_1 and Psoriasis_2). In psoriasis samples, affected areas exhibit strong S100A9 staining, whereas the healed areas show much weaker staining on the same slide. Enlarged boxed regions are shown on the left. Healthy skin samples for S100A9 and control IgG staining are displayed in the lower panels, both showing faint signals. Bars = 1000 μm and 250 μm. (**c)** Genome Browser tracks on human *IL36G* loci, showing chromatin accessibility peaks in disease-state (denoted as Pso-affected), clinically healed (denoted as Pso-healed), and control (denoted as control) keratinocytes, derived from merged samples for each condition. ChIP sequencing for H3K27Ac in primary human keratinocytes is also shown. (**d)** Immunohistochemistry for IL-36g and isotype control in psoriasis-affected and psoriasis-healed lesions (upper panels: Psoriasis_1 and Psoriasis_2). In psoriasis samples, affected areas exhibit strong IL-36g staining, whereas the healed areas show weaker staining on the same slide. Enlarged boxed regions are shown on the left. Healthy skin samples for IL-36g and control IgG staining with faint signals are displayed in the lower panels. Bars = 1000 μm and 250 μm. (**e)** Genome Browser tracks of murine keratinocytes at the *S100a9* and *S100a7a* loci in the imiquimod-induced psoriasis model. The tracks demonstrate an increase in chromatin accessibility at day 6 in the inflammatory phase, followed by a return to control levels at day 30 in the healed phase. Data are derived from published data (Naik et al, 2017). ChIP, chromatin immunoprecipitation.

The chromatin accessibility in clinically healed keratinocytes suggests a state of epigenetic priming; however, it remains unclear whether this accessibility translates into active protein expression. To address this issue, we examined the protein expression of *S100A9*, a representative gene within persistently accessible chromatin regions, using skin tissue sections from the same individuals, including psoriasis lesions, marginal regions, and clinically healed skin. We first confirmed a marked increase in chromatin accessibility at the promoter regions of *S100A9* in psoriasis-affected keratinocytes compared with that in keratinocytes of control skin, and the increased accessibility was maintained in paired psoriasis-healed keratinocytes (Figure 4a). Immunohistochemical staining showed marked S100A9 expression in the lesional and marginal epidermis. In contrast, much weaker staining was observed in the healed epidermis, with expression levels comparable with those in control healthy skin (Figure 4b). These tissue sections included contiguous lesional, transitional, and healed areas on the same slide, allowing internal validation of staining specificity under identical conditions. To further assess whether this uncoupling between chromatin accessibility and protein expression extended to other immune-related genes, we examined *IL36G*, which also exhibited persistent chromatin accessibility in clinically healed keratinocytes (Figure 4c). Consistent with previous reports, IL-36γ expression was observed predominantly in the upper epidermal layers of psoriatic lesions (Johnston et al, 2017). Staining in clinically healed skin was weaker, with reduced signal, particularly in the upper epidermis (Figure 4d).

Prior studies using the imiquimod-induced psoriasis model have delineated postinflammatory chromatin profiles in murine keratinocytes, identifying sustained chromatin accessibility at the *Aim2* locus after inflammation resolution (Fernandes-Alnemri et al, 2009; Guo et al, 2015; Naik et al, 2017). Notably, in this transient mouse psoriasis model, the chromatin accessibility at *S100a* genes—which were highly accessible in human healed keratinocytes in our study—was strongly induced during the peak of inflammation but reverted to near-control levels by day 30 after inflammation resolution (Naik et al, 2017) (Figure 4e). These results revealed an apparent dissociation between chromatin accessibility and protein expression in clinically healed keratinocytes. Despite the existence of an epigenetically primed state, as indicated by chromatin accessibility at promoter loci, the lack or reduction of protein expression suggests that these cells remain transcriptionally poised but do not actively translate these transcripts under resting conditions.

### Distinct pathways associated with chromatin accessibility recovery rates in clinically healed epidermal keratinocytes

**Figure 5:**
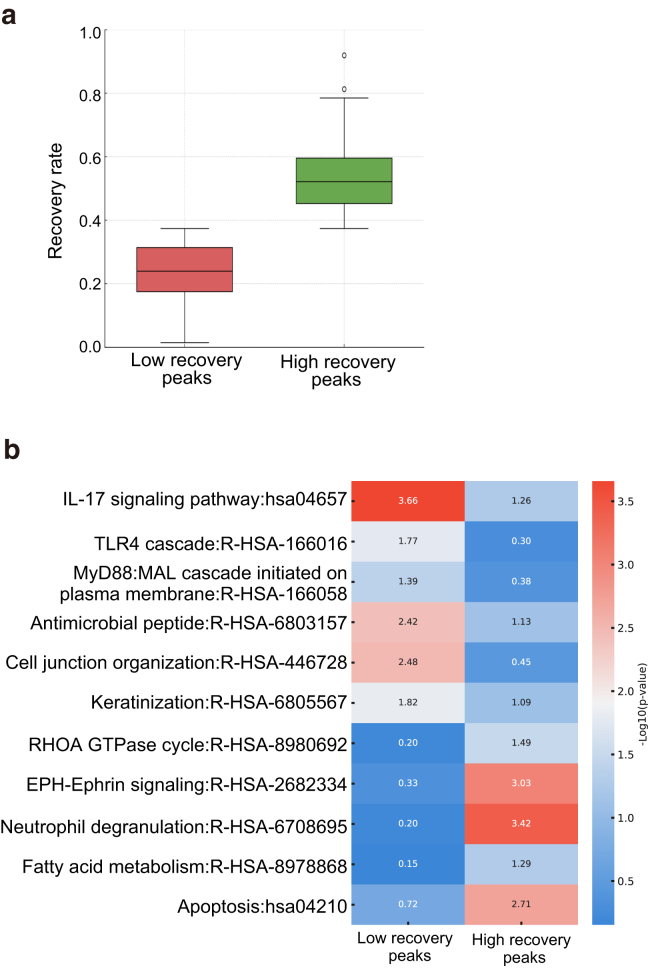
**Distinct pathways associated with chromatin accessibility recovery rates in clinically healed epidermal keratinocytes.(a)** Box plot showing the recovery rates of chromatin accessibility peaks categorized into 2 groups: low-recovery peaks and high-recovery peaks. Recovery rates were calculated on the basis of chromatin accessibility changes in psoriasis-affected keratinocytes and their subsequent recovery in clinically healed keratinocytes. (**b)** Heatmap showing pathway enrichment analysis for genes associated with low-recovery peaks and high-recovery peaks. Selected pathways with the largest enrichment differences, relevant to psoriasis and keratinocyte biology, and with at least 1 group showing statistical significance (*P* < .05) are displayed.

Our next objective was to determine genes or pathways that exhibit a higher propensity for chromatin accessibility recovery and those that remain resistant to recovery within clinically healed keratinocytes. To investigate this further, we calculated recovery rates for individual chromatin peaks induced in disease-state psoriasis-affected keratinocytes and categorized them into 2 groups: high-recovery and low-recovery peaks (Figure 5a). Representative genes for low-recovery peaks included *IFI27*, *MIR21*, *S100A9* and *S100A7*, whereas those for high-recovery peaks included *SERPINB3*, *SLC38A1*, and *SLC43A2*. Functional enrichment analysis revealed distinct pathway signatures for the high- and low-recovery groups. Pathways with the largest enrichment differences, selected for their relevance to psoriasis and keratinocyte biology, were depicted in the heatmap (Figure 5b). Genes associated with low-recovery peaks showed enrichment in IL-17, MyD88, and toll-like receptor signaling; antimicrobial peptide production; cell junction organization; and keratinization, reflecting their roles in inflammation and keratinocyte differentiation. Genes associated with high-recovery peaks were enriched in pathways related to RHOA GTPase activity, EPH–Ephrin signaling, neutrophil degranulation, fatty acid metabolism, and apoptosis, indicating their involvement in cytoskeletal remodeling and metabolic processes. These findings reveal that chromatin accessibility recovery rates in clinically healed keratinocytes are not uniform but rather locus specific, reflecting the functional roles of the associated genomic regions.

## Discussion

This study provides a cell type–specific perspective on chromatin accessibility in psoriasis-healed keratinocytes, demonstrating that clinically healed keratinocytes retain a unique chromatin landscape distinct from both disease state and healthy skin. Epigenetic landscapes are inherently cell-type specific, and thus, bulk analyses of whole skin may obscure cell-intrinsic regulatory mechanisms. By focusing on keratinocytes, our study provides a refined view of epidermal chromatin remodeling, highlighting how postremission regulatory states are maintained at the cellular level. Interestingly, the principal component analysis revealed tighter clustering of psoriasis-affected and psoriasis-healed keratinocytes than healthy controls, suggesting that psoriatic inflammatory conditions may constrain epigenetic heterogeneity. Although many disease-state–associated chromatin accessibility changes were not observed in healed keratinocytes compared with that in healthy skin, specific loci, including *S100A7, S100A8, S100A9,* and *IL36G*, exhibited increased accessibility, suggesting a transcriptional potential that may affect future inflammatory responses. Although direct comparison between psoriasis-affected and psoriasis-healed keratinocytes did not reveal statistically significant global differences in chromatin accessibility, this likely reflects the biological similarity between paired samples from the same individuals. Nevertheless, the fact that both psoriasis-affected and psoriasis-healed keratinocytes differed markedly from healthy control samples indicates that clinically healed skin retains disease-associated chromatin features. This finding supports the concept of a residual epigenetic state that persists even in the absence of visible inflammation and may contribute to site-specific disease recurrence.

Prior studies using the imiquimod-induced psoriasis model have delineated postinflammatory chromatin profiles in murine keratinocytes, identifying sustained chromatin accessibility at the *Aim2* locus after inflammation resolution (Fernandes-Alnemri et al, 2009; Guo et al, 2015; Naik et al, 2017). Notably, in this transient mouse psoriasis model, the chromatin accessibility at *S100a* genes—which were highly accessible in human healed keratinocytes in our study—was strongly induced during the peak of inflammation but reverted to near-control levels by day 30 after inflammation resolution (Naik et al, 2017) (Figure 4e). These findings suggest a fundamental difference between human psoriasis and transient inflammatory responses in mice. Human psoriasis is characterized by prolonged disease duration, recurrent flares, and patient-specific factors that may contribute to sustained chromatin remodeling. Nevertheless, given that AIM2 and S100As are activated in response to stress stimuli (Fernandes-Alnemri et al, 2009; Guo et al, 2015; Jonasson et al, 2017; Schonthaler et al, 2013; Vogl et al, 2018; Wang et al, 2018), our findings suggest that inflammatory persistence in keratinocytes may be associated with stress-responding regulatory programs in both mice and humans.

This study further investigated the relationship between chromatin recovery rates and associated genes and pathways in clinically healed keratinocytes. Low-recovery peaks, associated with genes such as *S100A*s and IFN alpha-inducible factor *IFI27*, demonstrated sustained chromatin accessibility in pathways central to inflammatory responses (Jonasson et al, 2017; Schonthaler et al, 2013). These regions may serve as epigenetic reservoirs, enabling rapid transcriptional activation upon subsequent inflammatory stimuli and contributing to proper immune responses. However, this responsiveness, if excessively activated, could increase the risk of dermatitis recurrence or chronic inflammation. In contrast, high-recovery peaks were associated with genes enriched in pathways related to cytoskeletal remodeling and metabolic regulation (Lim et al, 2012), suggesting that chromatin accessibility at these loci normalizes after healing, potentially contributing to epidermal homeostasis. This selective regulation may be crucial for maintaining a balanced tissue microenvironment after healing.

Another finding of this study is the association between chromatin accessibility and SEs. In psoriasis-affected keratinocytes, increased chromatin accessibility was prominent in SE regions, which are characterized by dense transcription factor occupancy and its regulatory activity (Blobel et al, 2021; Chen et al, 2020; Hnisz et al, 2013; Pott and Lieb, 2015). SE accessibility may thus contribute to disease-specific transcriptional programs and stable disease pathology. Previous studies have shown that NF-κB directs dynamic SE formation by recruiting BRD4, a member of the bromodomain and extraterminal domain family, in inflammatory endothelial cells and that BRD4 depletion impedes this SE reorganization (Brown et al, 2014). Moreover, NF-κB signaling directs changes in enhancer landscapes, with H3K27ac deposition stabilizing inflammatory enhancer activity (Borghini et al, 2018), highlighting the critical role of SEs in the pathogenesis of inflammatory conditions associated with NF-κB activation.

In summary, this study shows that clinically healed psoriatic keratinocytes retain a distinct chromatin landscape differing from both affected and healthy skin that may facilitate rapid immune activation upon subsequent stimuli. Certain *S100A* genes identified in our study have previously been reported to show altered expression in nonlesional psoriatic skin. However, whether the chromatin accessibility changes observed in healed keratinocytes reflect an epigenetic memory of prior inflammation or a healing-specific chromatin state remains unclear. At present, this distinction is unresolved, and direct comparisons with never-inflamed keratinocytes are needed in future studies. In addition, elucidating the histone modifications landscape of healed keratinocytes may provide more specific insights into targeted interventions that modulate residual chromatin changes and reduce the risk of psoriasis relapse.

## Materials and Methods

### Study design and sample collection

In this study, we examined the chromatin landscape of epidermal keratinocytes from psoriatic affected skin and adjacent healed skin samples, comparing them with samples from individuals without inflammatory skin disease. Our cohort consisted of 17 skin samples: 12 samples of paired affected and healed areas from 6 patients with psoriasis and 5 normal skin samples from 5 control participants. Nonexposed skin areas were sampled to eliminate potential confounders, such as UV light exposure. Clinical information, including patient age, sex, biopsy site, and treatment history, is provided in Table 1.

**Table 1:** Clinical Information of Study Participants

Group	Age, y	Sex	Body Site	Treatment History
Psoriasis	55	M	Abdomen	Steroid, vitamin D3 analog
Psoriasis	73	M	Thigh	Steroid, vitamin D3 analog
Psoriasis	27	M	Thigh	Combination ointment of calcipotriol and betamethasone dipropionate
Psoriasis	48	M	Thigh	Combination ointment of calcipotriol and betamethasone dipropionate
Psoriasis	31	M	Back	Combination ointment of calcipotriol and betamethasone dipropionate
Psoriasis	47	F	Back	Combination ointment of calcipotriol and betamethasone dipropionate
Control	34	M	Back	—
Control	65	M	Back	—
Control	45	F	Abdomen	—
Control	58	M	Abdomen	—
Control	74	M	Back	—

Abbreviations: F, female; M, male.

For accurate comparisons, affected skin samples were taken from an area 1 cm inside the margin of the psoriasis rash, and healed skin samples were taken from an area 1 cm outside the margin. The healed area was specifically chosen from sites with a history of healing and was visually confirmed to be free of skin rash other than pigmentation. Control skin was collected from normal skin discards after surgery of patients with no history of inflammatory skin diseases.

All participants provided written informed consent. The medical ethics committee of University of Tokyo approved all described studies (number 2022272NI), and the study was conducted according to the principles of the Declaration of Helsinki. All participants were Asian, and no further racial or ethnic subgrouping was performed because the study did not aim to analyze differences by race or ethnicity.

### Sample preparation

The epidermal layer was isolated from the collected skin samples by floating on 500 PU/ml Dispase (Godo Shusei, Tokyo, Japan) treatment at 4 °C overnight. After this, the epidermal layer was incubated with 0.25 % trypsin (Gibco, Waltham, MA) for 10 minutes at 37 °C to obtain single-cell suspension. CD45-positive immune cells in the epidermis were depleted using a negative selection process by Mojosort Human CD45 Nanobeads (BioLegend, San Diego, CA) for further ATAC-seq analysis (Figure 1b).

### ATAC-seq and data analysis

The construction of cDNA libraries for ATAC-seq was performed as described (Corces et al, 2017). A total of 5–20 × 10^3^ fresh keratinocytes were resuspended in lysis buffer (10 mM Tris-hydrogen chloride at pH 7.4, 10 mM sodium chloride, 3 mM magnesium chloride, 0.1% NP-40, 0.1% Tween 20, 0.01% Digitonin) for 3 minutes on ice and centrifuged in wash buffer (10 mM Tris-hydrogen chloride at pH 7.4, 10 mM sodium chloride, 3 mM magnesium chloride , 0.1% Tween 20) for 10 minutes at 4 °C. Pellets were resuspended in a 25-ml reaction buffer with 5 ml Tn5 (Illumina Tagment DNA Enzyme and Buffer Small Kit, Illumina, San Diego, CA) and 0.01% Digitonin and incubated at 37 °C for 30 minutes. Transposed DNA was amplified and purified using magnetic beads (AMPure XP, Beckman Coulter, Brea, CA). The quality and DNA concentration of libraries were evaluated using Bioanalyzer (Agilent, Santa Clara, CA). Libraries were sequenced using an Illumina NovaSeq 6000 platform. After removing adapter sequences and trimming reads, the read alignment was performed on the hg19 assembly using Bowtie2 genome alignment algorithm 1.4.1 (Langmead et al, 2009). Duplicate reads were removed by PICARD Tools (Broad Institute). Peak calling for ATAC-seq was performed with MACS2 using the following parameters for peak calling: --keep-dup all --nomodel --shift 37 --extsize 73 (Zhang et al, 2008). The BED files for all samples were merged into a unified peak set using BEDTools ‘merge’ command (Quinlan and Hall, 2010). FeatureCounts tools (Liao et al, 2014) were then utilized to quantify the reads mapping to each peak across all samples. The obtained read counts were normalized and analyzed for differential accessibility using DESeq2 (Love et al, 2014). To visualize the results, heat maps of genes associated with differentially accessible chromatin sites were generated using iDEP, an open-source software for integrated differential expression and pathway analysis (Ge et al, 2018). Pathway analysis was also performed with iDEP. GSEA was conducted using the GSEA software (version 4.3.3) and the MSigDB (Molecular Signatures Database) (version 2023.2) (Liberzon et al, 2011; Subramanian et al, 2005). The enrichment score and normalized enrichment score were calculated, with significance determined using a false discovery rate q-value cutoff of 0.25. In addition, GSEA was utilized to analyze whether genes associated with differentially accessible chromatin sites were significantly enriched in specific gene sets related to typical enhancers or SEs under in vitro keratinocytes stimulated with cytokines. Homer de novo motif discovery algorithm was used for motif discovery analysis of differential Assay for Transposase-Accessible Chromatin peaks (Heinz et al, 2010). Venn diagrams were generated with the online-based Venny Tool.

### Chromatin immunoprecipitation sequencing and data analysis

Chromatin immunoprecipitation was performed as previously described with some modifications (Shibata et al, 2020). Cells were fixed with 1% fresh formaldehyde for 8 minutes at room temperature and then quenched by 2.5 M glycine. Cells were lysed in swelling buffer (25 mM 4-[2-hydroxyethyl]-1-piperazineethanesulfonic acid potassium hydroxide at pH 7.8, 1.5 mM magnesium chloride, 10 mM potassium chloride, 1% igepal, 1 mM dithiothreitol, 1x protease inhibitor cocktail) for 10 minutes on ice, followed by dounce homogenization (Activ motif, Carlsbad, CA). Nuclei were pelleted and resuspended in RIPA buffer (10 mM Tris at pH 8.0, 140 mM sodium chloride, 1% Triton X-100, 0.1% sodium deoxycholate, 0.1% SDS, 1 mM EDTA, 1x protease inhibitor cocktail). Chromatin was sonicated to an average size of 300–500 bp with the Diagenode Bioruptor (Diagenode, Seraing, Belgium). Sheared chromatin was immunoprecipitated overnight at 4 °C with the antibody against H3K27Ac (Abcam, Waltham, MA) that was prebound to Dynabeads Protein G (Thermo Fisher Scientific, Waltham, MA). After washing and elution, chromatin was de-cross-inked overnight at 65 °C and purified using the DNA Clean and Concentration 5 kit (Zymo Research, Irvine, CA). The construction of cDNA libraries for chromatin immunoprecipitation sequencing was performed using the NEBNext UltraII DNA PCR-free Library Prep Kit for Illumina (New England Biolabs, Ipswich, MA), according to the manufacturer’s instructions. Libraries were sequenced using an Illumina NovaSeq 6000 platform. Read alignment was performed on hg19 assembly of the human genome using the STAR genome mapper with the option to disable spliced alignments and prohibit gaps (Dobin et al, 2013). Peak calling for chromatin immunoprecipitation sequencing was performed using the Homer findpeaks algorithm with –style histone or –style super parameters (Heinz et al, 2010).

### Immunohistochemistry

Immunohistochemistry staining was performed on formalin-fixed, paraffin-embedded skin sections. Sections were deparaffinized and then used for antigen retrieval using a citrate-based buffer (Vector Laboratories, Burlingame, CA). Primary antibodies used for staining were S100A9 and IL36γ (Abcam), followed by detection with 3,3’-diaminobenzidine (Vector Laboratories). For negative controls, species- and isotype-matched IgG (rabbit IgG and mouse IgG1, Abcam) were used in place of the primary antibodies.

### Statistical analysis

Statistical analyses were performed using R and associated tools. Differential chromatin accessibility was assessed using DESeq2, and significant peaks were defined as those with adjusted *P* < .05 using Benjamini–Hochberg correction in Figures 2 and 3. GSEA was performed with GSEA software, using MSigDB, with significance defined at false discovery rate q < 0.25 in Figure 1. Fisher’s exact test was used for categorical enrichment comparisons, including peak distributions of SEs in Figure 2. One-way ANOVA followed by Sidak's multiple comparisons correction was used to assess differences in normalized signal intensities of each locus in Figure 3. Motif analysis was performed with HOMER, and transcription factor enrichment was assessed using the TRRUST module within Metascape in Figures 2 and 3. Principal component analysis was conducted using R’s prcomp function in Figure 3. Heatmaps in Figure 1 were generated using iDEP, whereas chromatin accessibility heatmaps and metaprofiles in Figure 3 were generated using deepTools. Recovery rates for differentially accessible peaks were calculated to quantify the degree of chromatin accessibility normalization in healed keratinocytes, and differences between low- and high-recovery groups were evaluated using boxplot visualization. Pathway enrichment analyses for each recovery group were performed using Kyoto Encyclopedia of Genes and Genomes (clusterProfiler) and Reactome (ReactomePA) with Benjamini–Hochberg correction for multiple testing in Figure 5.

## Ethics Statement

The study was approved by the medical ethics committee of the University of Tokyo (number 2022272NI). All participants provided written, informed consent in accordance with the Declaration of Helsinki.

## Data Availability Statement

Data are available from a public repository at the national DNA Data Bank of Japan database under the accession numbers JGAS000844 for the study and JGAD 000986 for dataset (https://www.ddbj.nig.ac.jp/index-e.html). The additional data that support the findings of this study are available from the corresponding author on reasonable request.

## ORCID

Sayaka Shibata: http://orcid.org/0000-0003-3382-8026

## Conflict of Interest

The authors state no conflicts of interest.
